# TTR amyloidosis: a scientific journey since Andrade

**DOI:** 10.1186/1750-1172-10-S1-I19

**Published:** 2015-11-02

**Authors:** Maria João Saraiva

**Affiliations:** 1 Instituto de Inovação e Investigação em Saúde (I3S), Universidade do Porto, Porto, Portugal; 2 Unidade de Neurobiologia Molecular, IBMC – Instituto de Biologia Molecular e Celular, Universidade do Porto, 4150-180 Porto, Portugal

## 

Mário Corino de Andrade described in 1952 in the journal “Brain” the first form of an hereditary amyloidosis, Famlial Amyloidotic Polyneuropathy, FAP - affecting the peripheral nervous system, also known as Andrade´s disease. In 1939 Andrade observed different patients complaining of loss of sensitivity to temperature and pain. These symptoms affected other families in the area, namely fisherman who complained of loss of sensitivity in their feet when touching the ropes of their small fishing boats. Andrade with the extraordinary intuition that all collaborators admired, realized the clinical symptoms deviated from what he had seen until then.

Andrade suspected he was in the presence of a rare hereditary disease. To understand in depth what he considered new, he asked the collaboration of experts in different fields, like genetics, pathology, confirming the genetic nature of the disease and the presence of systemic amyloid deposits, particularly in the peripheral nervous system. Since this seminal work, the explosion of molecular biology tools gave us today perspectives for therapies.

Some of the concepts and hypotheses put forward then with basic equipment and techniques available in the last century evolved tremendously giving us molecular and cellular in-depth knowledge; Andrade´s vision is still updated but need urgent clarification if we want to move towards urgent efficiency therapies of the disease. This lecture will exemplify one case where we still stand behind a black box despite access to modern know-how.

